# Design and prototype validation of a laterally mounted powered hip joint prothesis

**DOI:** 10.1177/20556683241248584

**Published:** 2024-04-30

**Authors:** Sarah Mroz, Natalie Baddour, Patrick Dumond, Edward D Lemaire

**Affiliations:** 1 Department of Mechanical Engineering, 6363 University of Ottawa, Ottawa, ON, Canada; 2 10055 Ottawa Hospital Research Institute, Ottawa, ON, Canada

**Keywords:** Robotic prosthetic devices, mechanical loading, design requirements, limb prosthetics, amputees

## Abstract

Prosthetic technology has advanced with the development of powered prostheses to enhance joint function and movement in the absence of native anatomy. However, there are no powered solutions available for hip-level amputees, and most existing hip prostheses are mounted to the front of the prosthetic socket, thereby limiting range of motion. This research introduces a novel laterally mounted powered hip joint (LMPHJ) that augments user movement. The LMPHJ is mounted on the lateral side of the prosthetic socket, positioning the hip joint closer to the anatomical center of rotation while ensuring user safety and stability. The motor and electronics are located in the thigh area, maintaining a low profile while transmitting the required hip moment to the mechanical joint center of rotation. A prototype was designed and manufactured, and static testing was complete by modifying the loading conditions defined in the ISO 15032:2000 standard to failure test levels for a 100 kg person, demonstrating the joint’s ability to withstand everyday loading conditions. Functional testing was conducted using a prosthesis simulator that enabled able-bodied participants to successfully walk with the powered prosthesis on level ground. This validates the mechanical design for walking and indicates the LMPHJ is ready for evaluation in the next phase with hip disarticulation amputee participants.

## Introduction

In the absence of native anatomy, prosthetic devices are often used to maintain mobility, independence, and quality of life for a person with a lower limb amputation. Hip disarticulation amputation (HDA), amputation through the hip joint, and hemipelvectomy amputation (HPA), amputation of part of the pelvic bone, are the highest levels of lower limb amputation, requiring prosthetic hip, knee, and ankle joints.^1,2^ Amputation level is highly correlated with reduced motor function and increased metabolic cost during ambulation,^3–5^ resulting in severe mobility issues for these high-level amputees. Prosthesis design is challenging since the device becomes more complex with more proximal amputations.

Hip-level amputees are met with large physical demands when using a prosthetic device. For this reason, approximately 43% of amputees abandon their prostheses shortly after being fitted,^4,6^ and instead, often opt for wheelchairs or arm crutches for ambulation. Those who use a prosthesis rely on lumbar spine movement for propulsion, accompanied by a heavier device than lower amputation levels, which imposes increased energy expenditure when walking.^4^ Gait patterns are typically highly asymmetrical, walking speed is reduced, and range of motion (ROM) is limited when walking with a prosthetic limb.^7^

Powered prosthetics are advancing towards a future where an artificial limb will mimic the biomechanical function it is replacing. However, prosthetic hip joints have not kept pace with these developments.^6,8^ Currently, most prosthetic hip joints on the market are mounted to the front of the prosthetic socket, positioned away from the anatomical joint centre. Commonly used devices include single-axis passive joints (e.g., Ottobock 7E7) and the more advanced Ottobock Helix^3D^. The Helix^3D^ polycentric hip joint with a hydraulic unit, when combined with a microprocessor-controlled knee joint, was found to improve amputee gait over previous devices.^9^ The joint utilizes replaceable tension springs that store energy during stance phase and release energy at the start of the swing phase, enabling faster hip flexion initiation.^10^ However, the Helix^3D^ is a passive joint that maintains a front-mounting orientation to the prosthetic socket. Without augmentation, amputees must generate hip movement through the pelvic and back muscles, which can lead to long-term injuries.^11^

Powered solutions are commercially available for knee and ankle joints, enabling walking mechanics closer to natural gait.^12–14^ These active systems have demonstrated improved mobility,^15^ increased symmetry,^12,16^ and a reduction of metabolic costs during walking.^17^ These positive findings suggest that powered prosthesis improve functionality for users. Therefore, opportunities exist to explore hip-level powered prosthesis designs. Powered assistance also presents the opportunity to relocate the joint centre of rotation closer to the anatomical hip centre since controls can enable safe actuation and provide a greater ROM. Providing concentric hip moments and a laterally mounted orientation will provide hip-level amputees with a prosthetic system that enables more natural hip movement during gait. The LMPHJ should promote more symmetrical loading across the hip joints and reduce user energy expenditure. Accompanied by a customized control system, the amputee should feel confident and comfortable using the device.

Mobility, independence, and quality of life for people with HDA and HPA should improve with more advanced biomimetic prosthetic designs. A more functional device can provide amputees with improved, safer mobility, and this technology can enable users to participate more effectively in their daily lives. The goal of this study is to design, manufacture, and validate a novel laterally mounted powered hip joint for hip-level amputees. This involves verifying the joint’s structural integrity, assessing joint functionality and usability, and conducting testing to validate effectiveness within a hip-knee-ankle-foot prosthesis.

## Methods

### Design requirements

The LMPHJ design requirements are based on available literature, existing technology, and well-accepted international standards to ensure the design adequately addresses user needs. Functional design criteria include factors required to enable users throughout daily activities. Available data from the literature and existing technology was used to define device requirements on range of motion, angular velocity, power, and torque. Non-functional requirements are based on user safety, manufacturing criteria, and anthropometric measurements to ensure the device does not geometrically exceed standard body proportions. The LMPHJ design requirements are:

• Joint range of motion of at least 130° flexion and 15° extension to enable daily movements such as walking, sitting, reaching, etc.^18^
• Joint angular velocities greater than or equal to 150°/s to accommodate variations in gait and faster walking speeds anticipated with improved gait^19–21^
• Joint accommodates users up to 100 kg^22^
• Peak hip moment requirement of 1.21 Nm/kg, translating to approximately 125.0 Nm for a 100 kg person^19,23^
• Maximum hip net joint power requirement of 360.0 W to provide sufficient augmentation during various daily movements such as inclines and stairs^24^
• As a first prototype, maximum joint weight of 30% more than the weight of the Össur Power Knee (2.7 kg), giving a maximum weight of 3.5 kg^25^
• No sharp edges for safety reasons
• Joint mounted to the lateral side of the prosthetic socket
• Joint should fit under clothing, not protruding laterally beyond natural width of hips.
• Joint must not interfere with sitting. Considering females in the 50^th^ percentile, thigh clearance is 12.9 cm. Posterior protrusion from axis of rotation ≤6.5 cm^26^
• Joint must not interfere with prosthetic socket through its range of motion. Motor positioned 13.0 cm distal to the axis of rotation^27^

### Design and development

**Figure 1.**
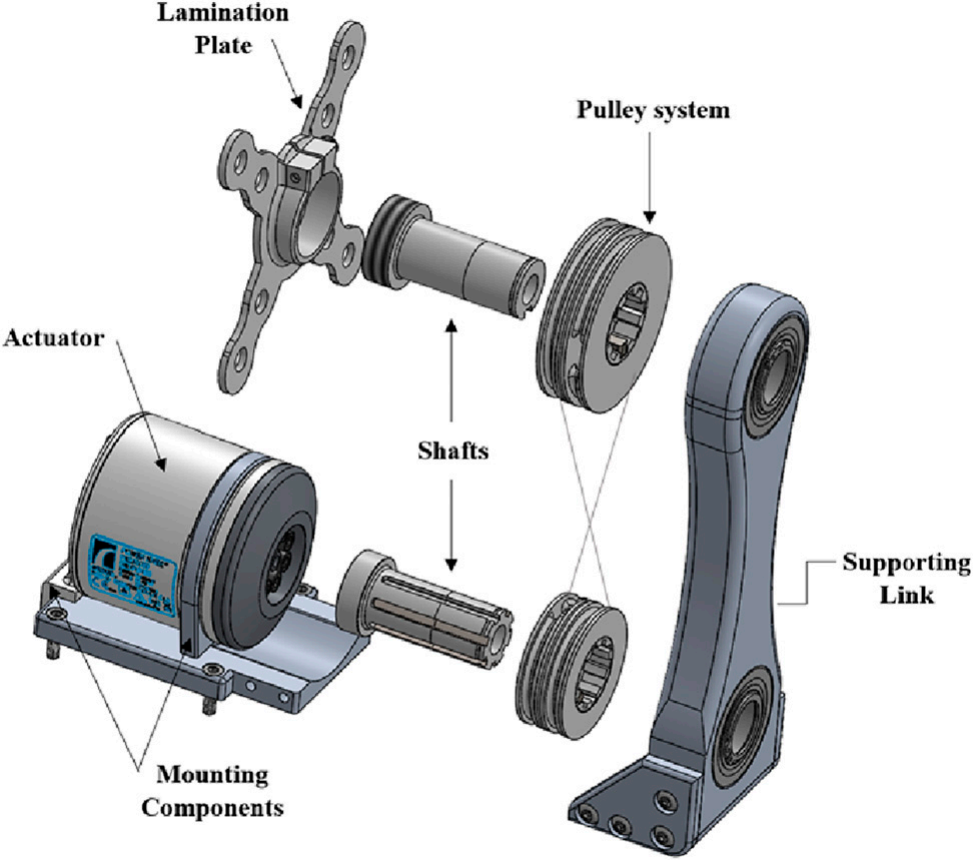
Main components of the laterally mounted powered hip joint design.

The new LMPHJ (Figure 1) is a mechanical system that actuates the prosthetic limb during gait to improve user mobility. More details on the design are available.^28^ The system includes:

• Pulley system: Drivetrain composed of two sets of pulleys in a crossed rope configuration to enable safe hip flexion and extension.
• Shafts: Structural components to support weight-bearing and actuation. The distal shaft interfaces with the motor and transmits the motion. The proximal shaft interfaces with the lamination plate, providing the axis of rotation.
• Supporting link: Structural element connecting the proximal and distal shafts, positioning the motor beneath the prosthetic socket.
• Lamination plate: Connecting component between the system and the prosthetic socket.
• Actuator: Össur Power Knee™ microprocessor-controlled motor modified for the LMPHJ design, providing sufficient torque and power to the system, and proven long-term performance in lower extremity prosthetics.
• Mounting components: Designed components for mounting the actuator and integrating the LMPHJ to the distal portion of the prosthetic limb.

This study focused on the mechanical aspects of the powered prosthesis. A customized control system is required but is beyond the scope of this paper. The design interfaces with an aluminum chassis located below the actuator that contains all electrical components and connections to a microprocessor-controlled knee joint. Considerations for electrical connections are included in the scope, but the chassis design was completed in parallel.

#### Mechanical drive system

**Figure 2.**
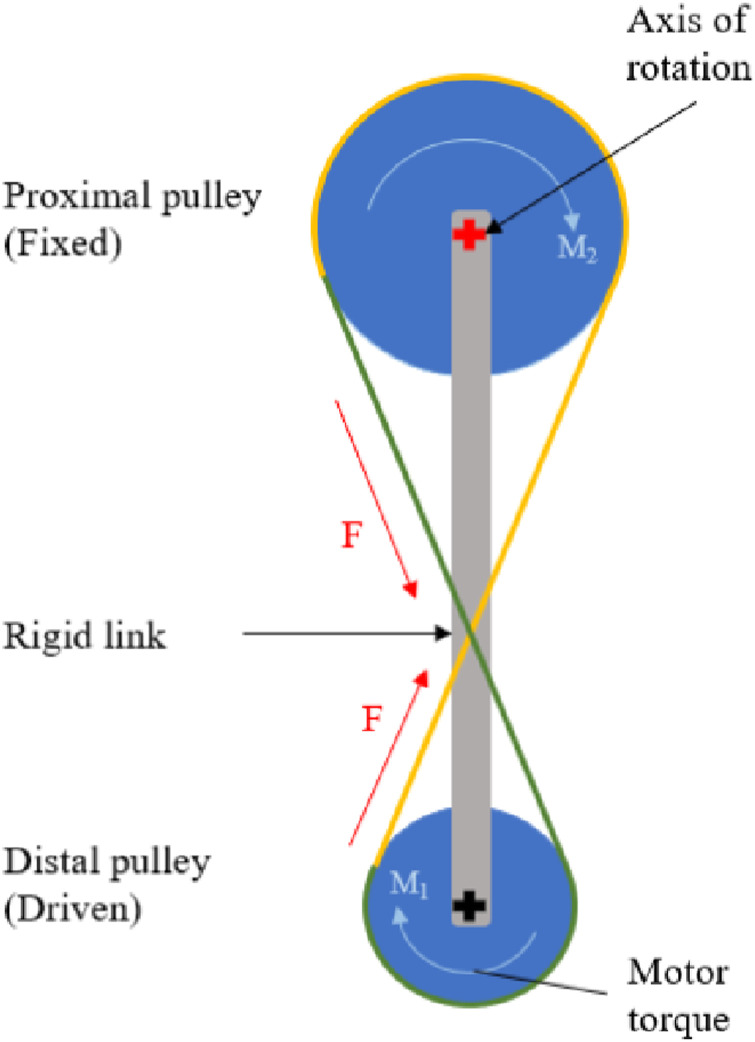
Pulley systems where F is tension in the ropes, M_1_ is motor torque, and M_2_ is reaction moment at the axis of rotation. Crossed rope concept for LMPHJ actuation, with dual pulley and rope system represented in green (Rope/Pulley 1) and yellow (Rope/Pulley 2).

Pulleys are used for the mechanical drive system. To achieve the intended functionality, the system includes two sets of custom-designed pulleys with two ropes in a crossed configuration (Figure 2). The top pulley is fixed relative to the socket, and the ropes are fixed relative to the pulleys. This configuration does not allow uncontrolled movement, which is essential for safety. Leg flexion or extension can only be achieved through motor actuation or back drive. Two sets of concentric pulleys are used, with one rope driving flexion and the other driving extension. The ropes must be crossed to enable the device to rotate about the top fixed pulley.

The Össur actuator specifications indicate a maximum motor torque of 96.0 Nm and a maximum speed of 300.0°/s. To satisfy both moment and speed requirements, a 0.7 torque ratio between the proximal and distal pulleys is required.

**Figure 3.**
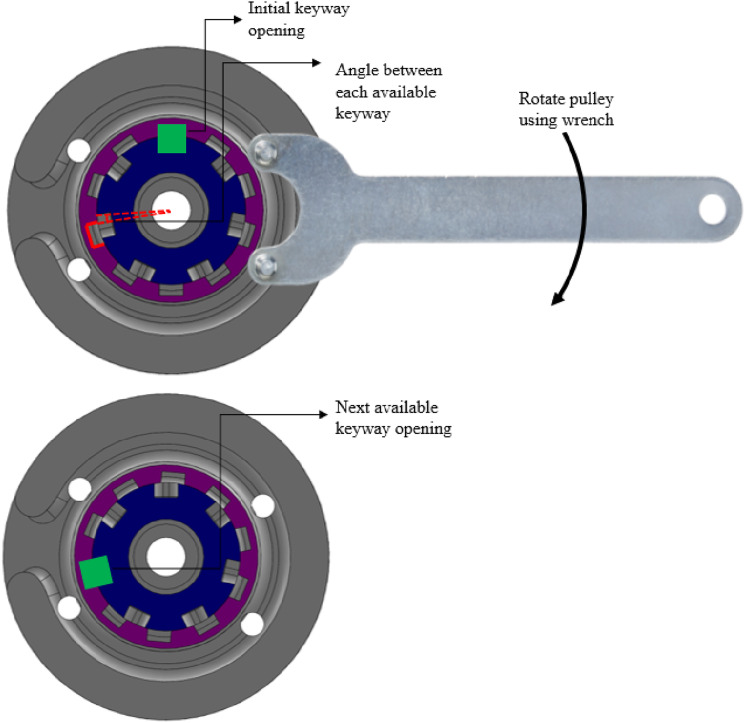
Keyway tensioning solution between the shafts (blue) and pulleys (purple) using an angle grinder wrench. The highlighted green key demonstrates various pulley positioning possibilities.

Vectran® rope was selected due to its strength to weight ratio, high tensile strength, and low stretch and creep resistant properties.^29^ The ropes must remain in constant tension because slack in the system can result in jerky movements that would not be tolerated by the user. A keyway solution was implemented to tension the system, with an odd number of keyways on the shaft and an even number on the pulleys offering angular positions every 9° (1.9 mm of rope) for 56 possible combinations (Figure 3).

#### Structural elements

A proximal shaft is located at the hip axis of rotation and interfaces with the lamination plate through a threaded connection and a pinch screw to prevent rotation. The distal shaft is integrated into the motor and positioned at its axis of rotation. These shafts are subject to high forces and fatigue loading; therefore, design decisions ensured that the shafts are strong enough, remain as lightweight as possible, and reduce bending moments and stress concentrations. High-strength steel 17-PH H900 is used for both shafts.

**Figure 4.**
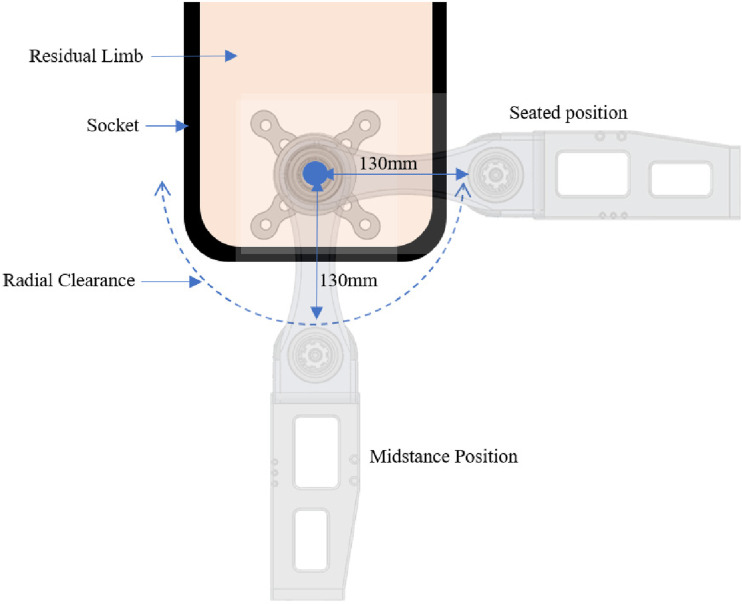
Radial clearance required to ensure no interference with the prosthetic socket throughout the ROM.

A supporting link between the proximal and distal shafts is required to facilitate rotation, provide structural support, and ensure adequate clearance between the motor and prosthetic socket through the full ROM. The supporting link is a critical weight-bearing component that endures extensive loading during use. The link is fabricated from Aluminum 2024 because it is lighter than steel alternatives and has relatively high strength. Each socket is unique to the user. However, the furthest distances from the center of rotation occur at the base or front of the socket. These distances govern the supporting link length to ensure clearance between the joint and socket. Additional space accounts for soft tissue and the prosthetic socket itself. For the initial prototype, a radial distance of 13 cm was used to ensure socket clearance for an average user during testing^27^ (Figure 4). The actuator geometry was considered, allowing future iterations to include an outer casing that encloses the joint. Rotation about each shaft is required to enable hip flexion and extension. Angular contact bearings were chosen to accommodate both axial and radial loads.

#### Socket interface

The lamination plate connects the LMPHJ to the prosthetic socket. The lamination plate design was adapted from existing pronged lamination plate designs.^30^ Since sockets vary between users, the prongs can be bent to the socket shape prior to lamination. The prong’s shape and holes allow lamination resin to penetrate and create a stronger hold. The standard M36 threaded connection for prosthetic applications is implemented to connect to the proximal shaft. A unique element of this design is the Q-angular feature. Naturally, the human femur is angled medially, allowing the leg to rotate about the acetabulum without colliding with the pelvic bone. This is referred to as the quadricep angle, or “Q-angle”, and has normative values of 17.0° for women and 12.0° for men.^31^ Aiding to improve symmetry across limbs, a 5.0° angle in the frontal plane is implemented to orient the prosthetic thigh segment medially. Only 5.0° are included in the angular feature since some users can have a smaller natural Q-angle. Further adjustments for each user can be accommodated in the socket design and lamination process.

#### Integration with the hip-knee-ankle-foot prosthesis

**Figure 5.**
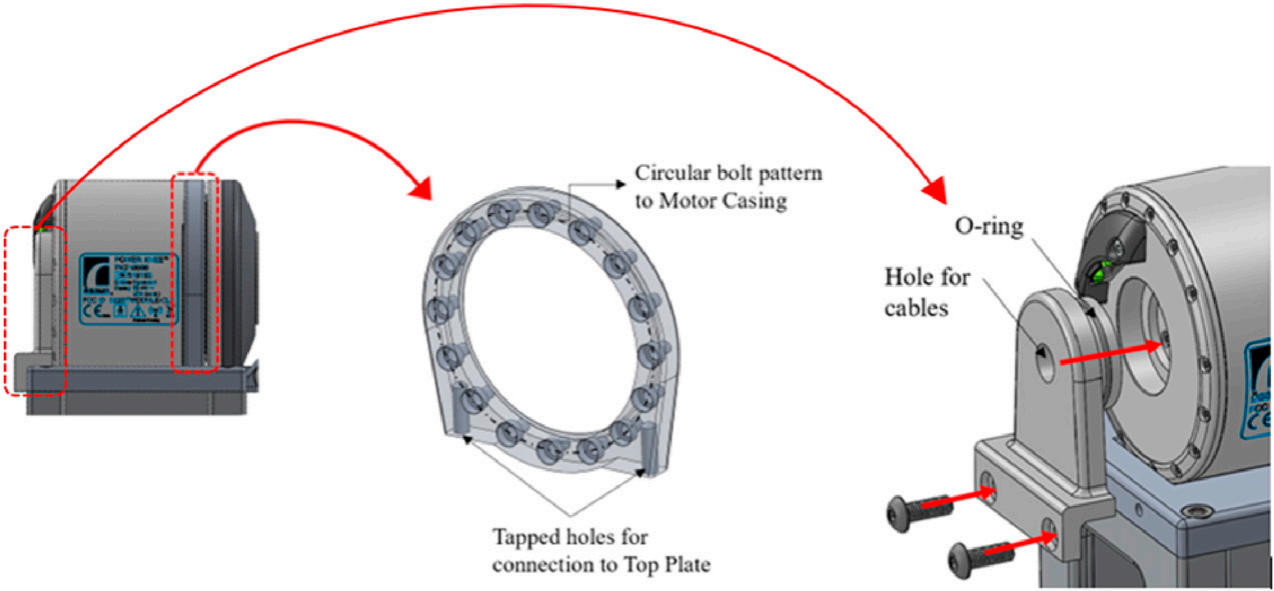
Motor mounting components (mounting ring and medial attachment component).

The Össur Power Knee actuator is used for this study. Several components were modified to integrate the actuator into the system. A motor mounting ring (Figure 5) was introduced to restrict motor movement relative to the device and ensure an appropriate axis of rotation alignment. The medial mounting part (Figure 5) was designed to be easily inserted into a usable cavity on the medial side of the motor. A rubber O-ring was implemented to ensure a snug fit and reduce the level of tolerancing required for machining. Together with the motor mounting ring, motor movement is restricted.

**Figure 6.**
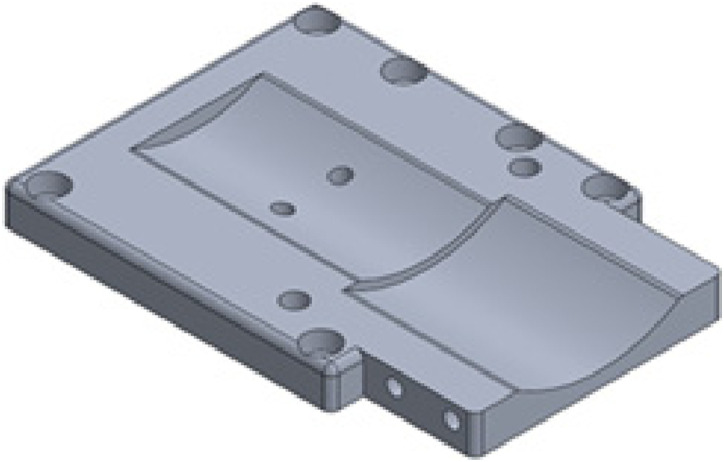
Top plate design for interfacing with the chassis.

The aluminum chassis that houses the electronics uses a top plate design (Figure 6) that matches the LMPHJ. An extension connects the supporting link and the chassis. A rigid connection between these parts is required to enable rotation. Otherwise, the distal shaft would rotate within the bearing connection without generating the desired motion. Grooves were added to accommodate the actuator’s circular dimensions. One groove is deeper than the other to provide clearance for the motor’s rotating components, avoiding contact between moving metal parts. Two holes were added on the bottom of the plate to secure the motor mounting ring, and tapped holes were added to the side for the medial mounting component. The overall plate thickness was designed to ensure the part is strong enough to support weight bearing during use.

### Prototype

**Figure 7.**
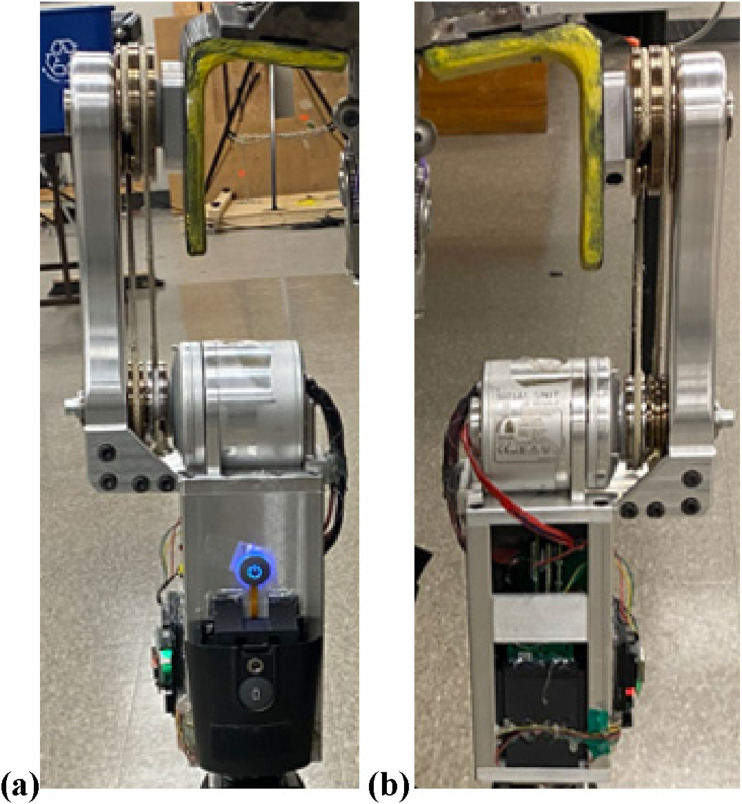
LMPHJ Prototype (a) front view, (b) back view.

A LMPHJ prototype was fabricated and assembled using a combination of machined and off-the-shelf components (Figure 7). To reduce fabrication time and since the LMPHJ was intended to be used with a prosthesis simulator for functional testing, not a prosthetic socket, a standard M36 adapter was used instead of the designed lamination plate. The mechanical system, including the actuator, weighs 3.5 kg and the electronics chassis weighs 2.2 kg, for a total system weight of 5.7 kg. The prototype validated the fabrication methods and assembly procedures.

## Testing and validation

### Static load testing

Mechanical testing objectives include ensuring LMPHJ structural integrity for walking and validating that the joint can withstand the loading required by international standards. Mechanical load testing was conducted at Össur’s facilities in Reykjavik, Iceland to validate LMPHJ structural integrity. Since load testing setups defined in the ISO-15032:2000 standard are based on front-mounted prosthetic hip joints,^22^ tests were modified to represent worst-case loading conditions for the lateral mounting orientation assuming the appropriate foot contact point, GRF line of action, and reference to the center of mass.

Anterior-posterior (AP) and medial-lateral (ML) tests were conducted with the joint in full extension to apply maximum loading conditions in the frontal and sagittal planes. Based on negligible stress and displacement results from computer simulations, torsional tests were not conducted. Additionally, cyclic testing was not in the scope of this study.

The reference planes and vertical offsets remained the same because they reference the position of the knee, hip, and other points relative to the ground. The coordinate system was adjusted relative to the criteria provided in the standard:

• Coordinate system origin at ground level, with distance from the ground to the knee reference plane of 500 mm.^32^
• u’-axis line extends from the origin and passes through the effective joint center defined by the middle of the proximal and distal shafts. Positive direction is upwards (in the proximal direction).
• o’-axis is perpendicular to the u’-axis and parallel to the hip joint centerline. Positive direction is outwards (in the lateral direction).
• f’-axis is perpendicular to both the u’ and o’-axis. Positive direction is towards the toe (in the anterior direction).

**Figure 8.**
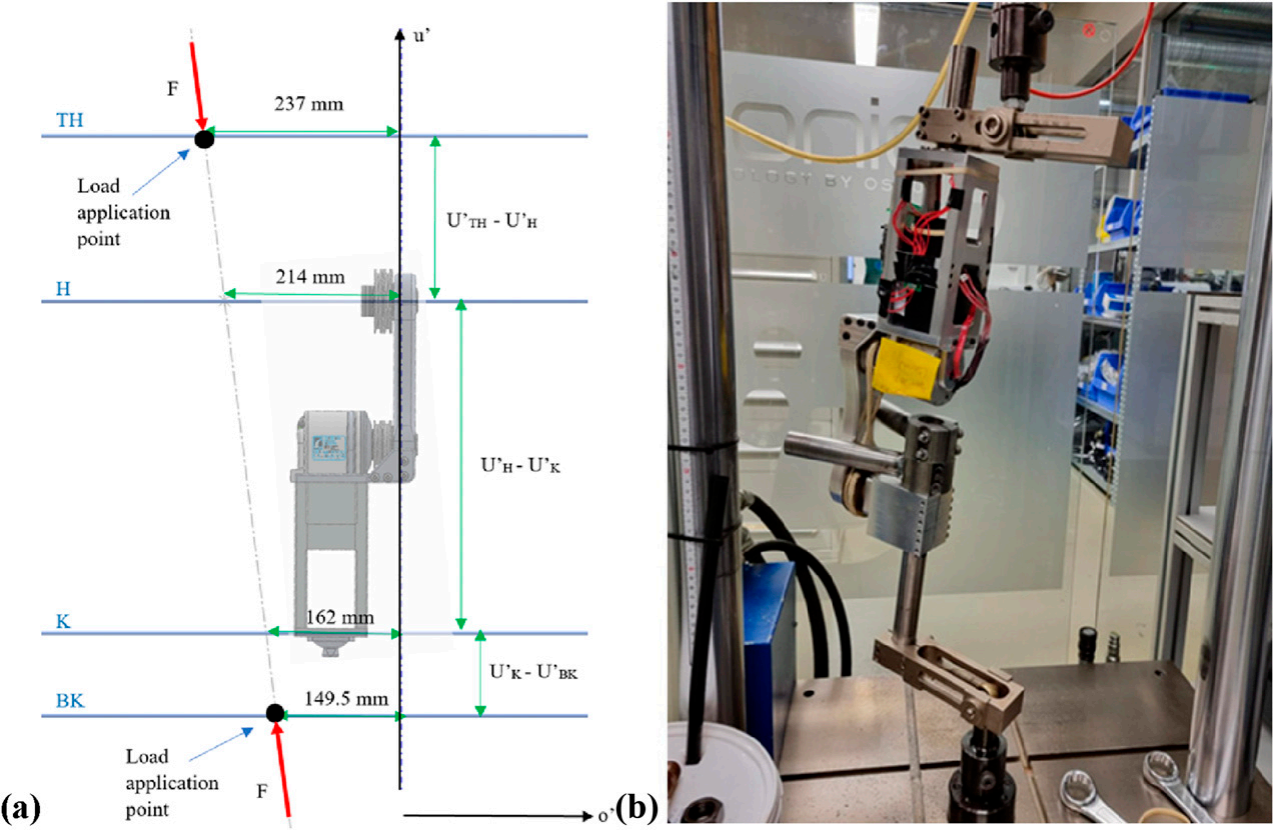
ISO-15032:2000 ML-extension test (a) TH = top reference plane for load application, BK = bottom reference plane for load application, H = hip reference plane, K = knee reference plane, (b) static load setup.

For the ML test, the line of application lies in the u’-o’ plane. Load line orientation was determined by assuming the center of mass (COM) is located at the center of the pelvis, and the foot contact point in the frontal plane would be in line with the anatomical hip joint center (Figure 8). The ground reaction force vector is directed from the foot contact point towards the body’s COM to maintain balance.^33^ A single compressive load is applied vertically; therefore, the joint was oriented in the machine at an angle to achieve the correct load line. For the ML test, the joint was positioned upside down to have the smaller lever arm closer to the load cell, decreasing the likelihood of damaging the joint.

**Figure 9.**
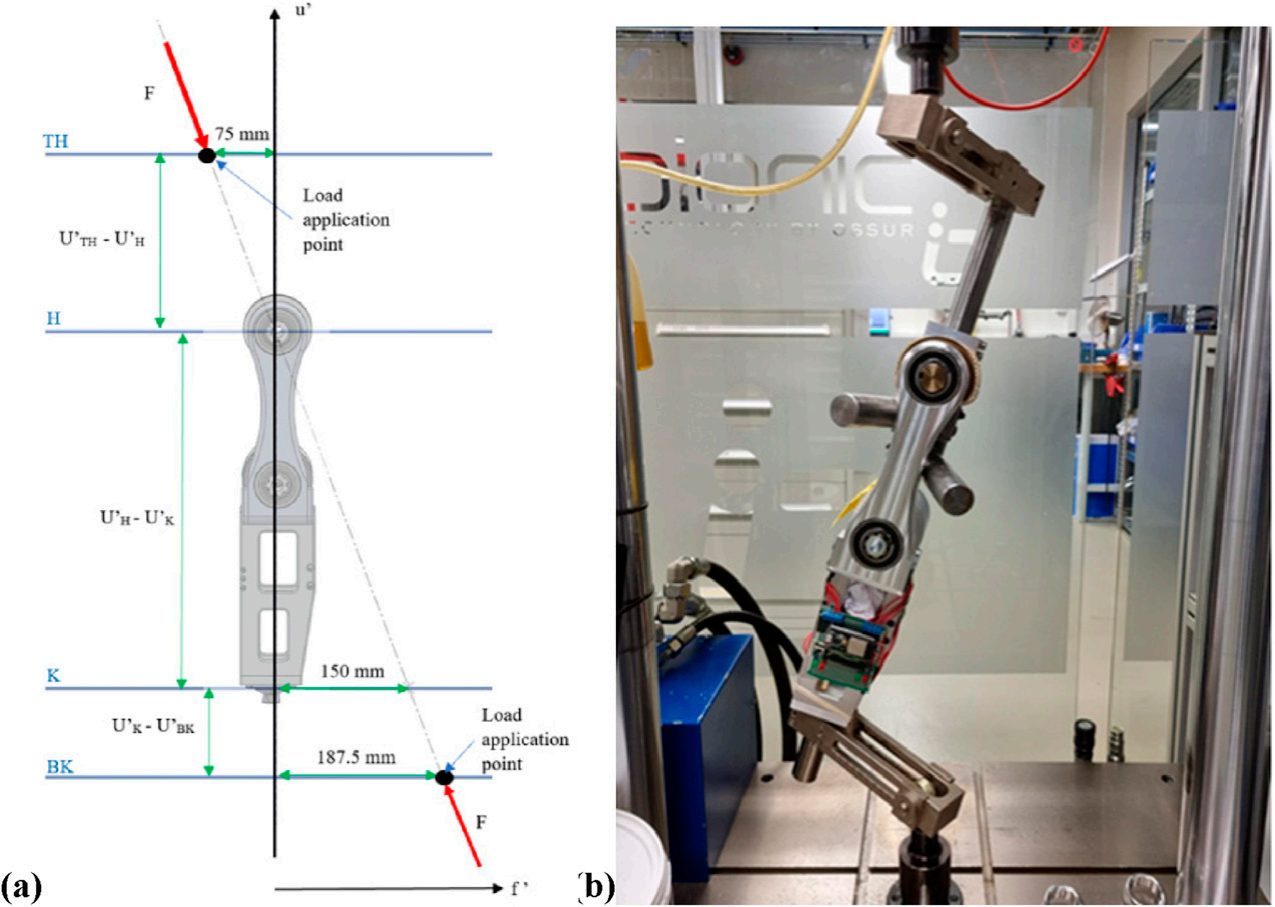
ISO-15032:2000 AP-extension test (a) TH = top reference plane for load application, BK = bottom reference plane for load application, H = hip reference plane, K = knee reference plane, (b) static load setup.

The AP test (Figure 9) occurs in the u’-f’ plane. For this condition, the load line orientation was maintained because the line of action is the same as for a front-mounted joint and, in this plane, the force can be easily translated to a known reference point. The line of action was relocated to pass through the effective hip joint center due to its proximity to the centre of mass in this plane. Again, the joint was loaded into the machine at an angle to achieve the correct load line.

Static load testing was conducted using a Servohydraulic Dynamic Low Force Testing System (Model LFV-5-ME, ID 4766) from walter+bai.^34^ Industry standard attachments were used to secure the LMPHJ into the testing machine. Mechanical stops were introduced to prevent joint rotation during testing.

A static proof test, followed by a static failure test, were completed for each condition, assuming the ductile failure criteria. The procedure for each test was as follows:

1. Prepare and align the test sample following the modified loading conditions
2. Apply a 1024 N settling force for approximately 30 s and then remove the force
3. Apply a 50 N stabilizing force
4. Increase the applied load incrementally to the test force at a rate of 200 N/s:
  o Proof test: 2240 N
  o Failure test: 3360 N (or until the sample fails)
5. Decrease the load at 200 N/s to the stabilizing force of 50 N
6. Zero the load and remove the LMPHJ from the testing machine
7. Inspect the sample for physical or functional defects

A displacement limit of 20 mm for all tests was specified to reduce the risk of severely damaging the joint if failure occurred. During testing, the load was incrementally applied until either limit was achieved, at which the machine began unloading the joint. Time, applied force, and vertical displacement were recorded at 100 Hz.

### Functional testing

**Figure 10.**
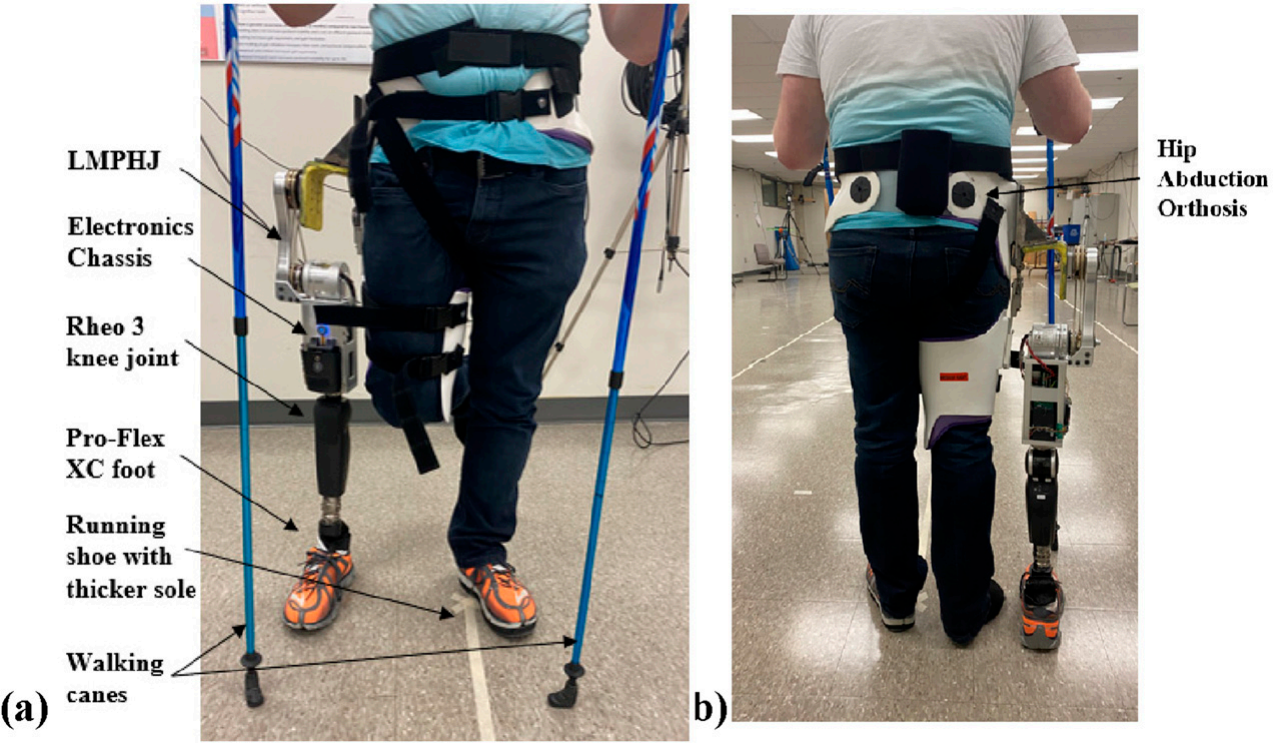
Test set-up for LMPHJ evaluation with able-bodied participants using hip prosthesis simulator (a) front view and (b) back view.

Functional testing was executed using a simulator that enables able-bodied participants to walk with a hip-knee-ankle-foot (HKAF) prosthesis.^35^ The test HKAF prosthesis (Figure 10) consisted of the LMPHJ, Össur Rheo 3 knee joint, and Össur Pro-Flex XC foot. A running shoe was worn on the prosthetic foot, and a modified running shoe with an insole 3 cm thicker was worn on the participant’s opposite limb. The thicker insole ensured the participant would have sufficient foot clearance on their non-loading limb (i.e., only the prosthesis and contralateral limb contacted the ground). The simulator was secured to the participant using a strap around the pelvic basket, two straps around the leg, and one underneath the right ischium. The option to use one or two walking canes was available to participants.

To assess walking with the LMPHJ, biomechanical parameters were evaluated to characterize each participant’s gait as a case series. A subjective comparison with the Helix^3D^ was made by participants regarding the LMPHJ functionality, since participants had previously trained on the HKAF prosthesis simulator with a Helix^3D^ hip joint and the same knee and foot components as this study. The test protocol was approved by the University of Ottawa Office of Research Ethics Board, ethics file number H-04-21-6811.

#### Participants

For enhanced safety, only able-bodied individuals were considered for preliminary functional testing with the simulator. Three participants (Table 1) volunteered to assess the LMPHJ’s functionality. Participants B and C used two canes for support, while Participant A was comfortable using only one cane. Each participant had previously learned to walk independently with the simulator using an OttoBock Helix^3D^ hip joint, Ossur Rheo knee joint, and Ossur Pro-Flex XC.^35^ Prosthetic leg length adjustments were required for each user to ensure approximately the same length as the contralateral side.

**Table 1.** Participant information.

	Participant A	Participant B	Participant C
Sex	Male	Male	Male
Age (years)	44	28	25
Height (cm)	178	180	175
Weight (kg)	95	95	98

#### Control system

**Figure 11.**
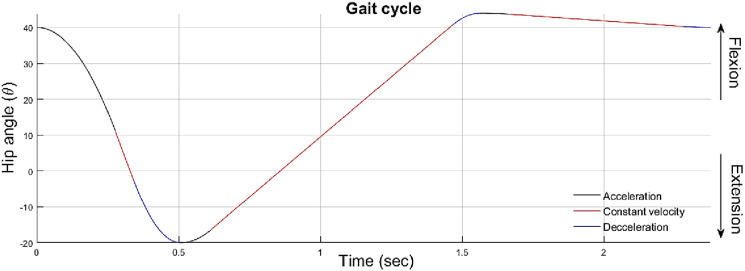
Gait profile used for LMPHJ functional testing.

The LMPHJ intelligent control system was under development at the time of the test. Therefore, a predetermined gait profile (Figure 11) was implemented to enable level ground walking using the simulator. The profile was uploaded to the LMPHJ control system, and the controls were tuned to achieve a comfortable gait pattern all users could follow. A relatively slow walking speed of 2.5 m/s was selected to ensure all participants could follow the prosthesis at this pace. The hip ROM was 45° maximum flexion and 20° maximum extension.

#### Data collection

Video data were recorded at 60 fps to extract spatiotemporal gait parameters throughout each test session. The investigator secured an iPhone 11 (2019) in a handheld tripod to assist with stability, positioned the phone approximately 1.5 m from the LMPHJ, and followed the participant throughout the test session while keeping the phone parallel to the prosthesis.

Eight walking strides were evaluated for each participant. The video data were visually analyzed to identify heel strike and toe off times. Kinovea^36^ was used to obtain hip joint angles throughout each test by tracking points identified in the video frames. The annotated videos were exported as MP4 files for reference, and the angular kinematics were exported as csv files for further analysis of hip ROM.

## Results

### Static load testing results

**Figure 12.**
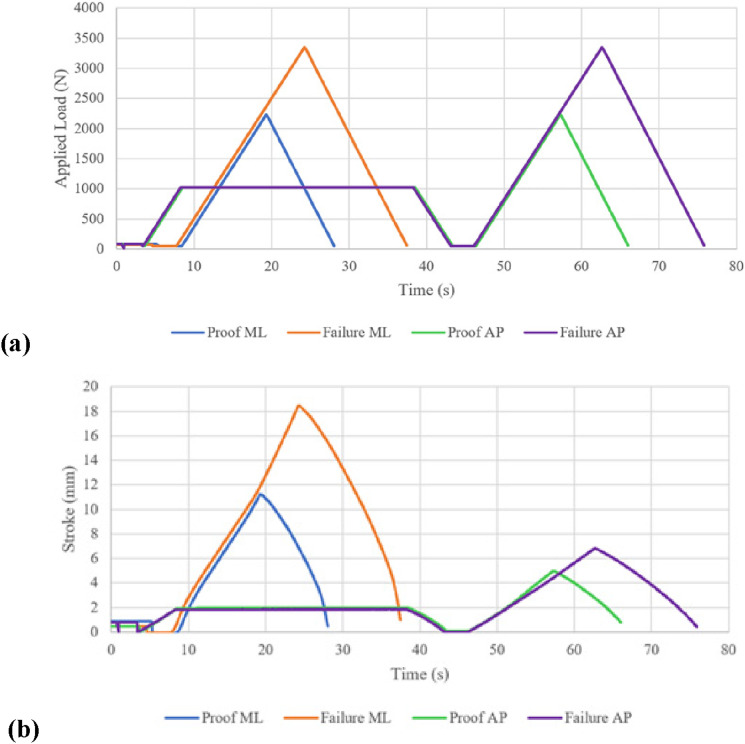
Loading profiles (a) static load tests and (b) vertical displacement results.

For each test, the loading profile was set to be the same following the procedure outlined in the Testing and Validation section. However, for the ML condition data collection was started late by the testing system operator and the 30 s settling force duration was not recorded. Therefore, in Figure 12, the peaks for each test occur at different times. The LMPHJ withstood the 3360 N ultimate test force, satisfying this element of the ISO standard failure criteria.

Vertical displacements recorded throughout the test were used to interpret LMPHJ deformation. Pearson correlations coefficients between the applied loads and displacements were computed for each test and resulted in correlation coefficients greater than 0.95 in all cases.

While the applied loads for each test were the same, displacements in the ML setup were much larger (Figure 12). During the ML test, a maximum displacement of 18.43 mm was recorded compared to 6.82 mm maximum displacement for the AP test.

Following mechanical testing, the LMPHJ was removed from the test rig and examined for any permanent deformation. No signs of failure or indications of functionality issues were observed.

### Functional testing results

All three participants successfully walked using the LMPHJ. For this pilot test, joint range was controlled by the actuator. The Össur actuator outputs a maximum angular velocity of 300.0°/s and a maximum torque of 96.0 Nm, corresponding to a speed of 220.0°/s and a torque of 130.9 Nm when considering the pulley size ratio. The maximum possible power generation for the system is the product of these parameters, resulting in 502.5 W peak power generation.

Table 2 presents the spatiotemporal gait parameters for each participant, obtained through 2D video analysis, averaged across eight gait cycles. Stride time was similar for all participants, with an average of 2.35 +/− 0.06 s. On average, the cadence for participants was 46.99 +/− 3.34 steps/min.

**Table 2.** Spatiotemporal gait parameters. Percentages (%) represent the proportion of average stride time.

Participant		A	B	C	Average
Stride time (s)		2.39 +/− 0.11	2.28 +/− 0.13	2.38 +/− 0.15	2.35 +/− 0.06
Step time (s)	Prosthetic	0.81 +/− 0.09 (33%)	0.98 +/− 0.10 (43%)	1.21 +/− 0.12 (52%)	1.0 +/− 0.20 (46%)
	Intact	1.58 +/− 0.14 (66%)	1.30 +/− 0.09 (57%)	1.16 +/− 0.05 (48%)	1.35 +/− 0.21 (57%)
Swing time (s)	Prosthetic	1.00 +/− 0.11 (42%)	0.83 +/− 0.05 (36%)	0.90 +/− 0.09 (38%)	0.91 +/− 0.09 (39%)
	Intact	0.38 +/− 0.09 (16%)	0.25 +/− 0.05 (11%)	0.29 +/− 0.06 (12%)	0.30 +/− 0.06 (13%)
Double Support (s)		1.00 +/− 0.12 (42%)	1.20 +/− 0.09 (53%)	1.19 +/− 0.16 (50%)	1.13+/− 0.10 (48%)
Cadence (steps/min)		44.15 +/− 2.00	46.16 +/− 2.9	50.67 +/− 2.90	46.99 +/− 3.34
Step time ratio		0.52	0.75	1.04	0.77

Step time variability occurred between participants for the prosthetic and intact limbs. Participant A had an average step time of 0.81 +/− 0.09 s on the prosthetic side and 1.58 +/− 0.14 s on the intact side, favouring the intact limb by 50% during stance. In comparison, Participant C achieved a similar average step time of approximately 1.2 s for both the intact and prosthetic limbs, demonstrating a high degree of step time symmetry.

The swing phase duration for all participants was distinctly longer for the prosthetic limb (0.91 +/− 0.09 s) than the intact limb (0.30 +/− 0.06 s). Additionally, participants were in double support for nearly 50% of the gait cycle.

**Figure 13.**
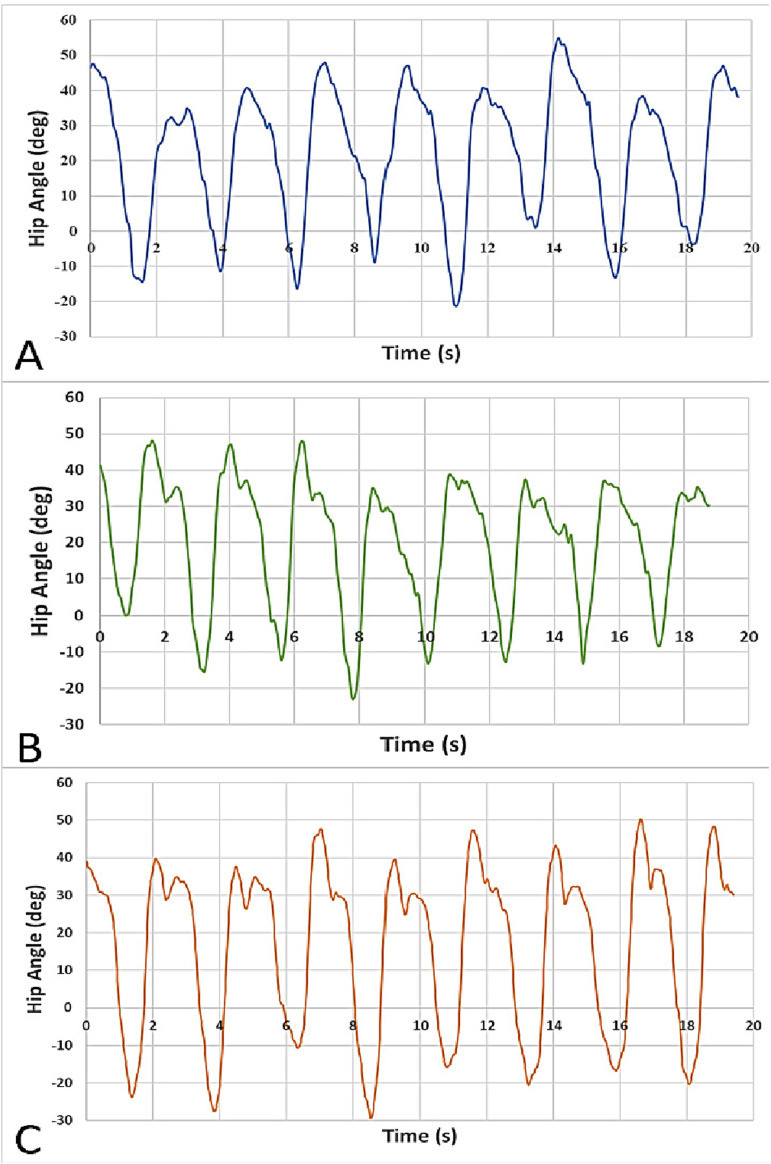
Hip angle (deg) versus time (s) of eight strides for Participant A, B, and C.

**Figure 14.**
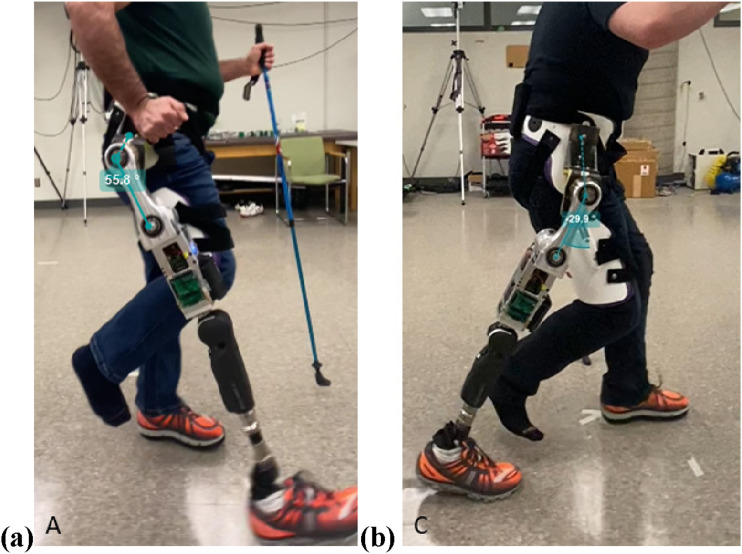
Maximum (a) hip flexion angle demonstrated by Participant A, and (b) hip extension angle demonstrated by Participant C.

The LMPHJ mean ROM was 58.31 +/− 5.12° with a maximum flexion angle of 51.14 +/− 3.43° and a maximum extension angle of 24.53 +/− 4.33° (Table 3). Figure 13 shows each participant’s ROM throughout the eight gait cycles. The maximum flexion and extension positions are demonstrated in Figure 14.

**Table 3.** LMPHJ range of motion averaged over eight gait cycles for each participant.

Participant	Maximum hip flexion angle (deg)	Maximum hip extension angle (deg)	Hip range of motion (deg)
A	54.89	−21.31	56.81 +/− 5.20
B	48.15	−22.84	64.95 +/− 3.45
C	50.38	−29.46	53.17 +/− 6.71
Average	51.14 +/− 3.43	24.53 +/− 4.33	58.31 +/− 5.12

When visually analyzing the videos, there were no drastic pelvic movements. Additionally, during testing sessions the participants expressed that they felt much less pelvic tilt was required to propel the prosthetic leg forward compared to previous sessions using the Helix^3D^ hip joint. Further research will be required to quantify these results.

## Discussion

### Discussion of design

LMPHJ mechanical and functional testing demonstrated the design successfully met all requirements, providing powered locomotion for hip-level amputees. The LMPHJ is designed to position the hip axis of rotation at its anatomical position and provide powered assistance to improve gait. The design criteria were based on biomechanical parameters from the available literature, existing technology, and international standards to determine an innovative solution that meets the needs of hip amputees. Functional and non-functional design requirements governed design decisions, to provide a mechanical system for improved performance, while maintaining safety and control throughout daily movements. The LMPHJ successfully met all design criteria in scope, but further development of the control system and design optimizations will be required.

The mechanical design (Figure 7) enables the full range of motion required for walking and other daily activities, with the lateral mounting allowing a much larger range of motion compared with front-mounted hip joints. For enhanced safety, a mechanical extension stop could be implemented in future versions to limit joint extension up to the limit of functional hip extension.

The LMPHJ weight was equivalent to the maximum criteria of 3.5 kg. Although the joint was relatively heavy, the actuation system was able to compensate for the weight and augment participant gait. Therefore, this did not affect LMPHJ functionality. The LMPHJ’s mechanical components, as well as electrical components and chassis, were all preliminary prototypes (Figure 7). As such, it should be possible to decrease overall weight and size in future design iterations via optimization. For example, only preliminary optimization was performed on the supporting link, with the prototype design being conservative to have a safe device for preliminary testing. Changing and removing material will produce a much lighter link component.

Geometric constraints were achieved so that the joint comfortably fits beneath pants, will not interfere with the prosthetic socket (Figure 4), and does not protrude in any direction beyond typical anthropometric measurements. Fillets were added to all exposed edges while modeling, and fabrication instructions specified the removal of sharp edges to ensure no risk of injury to the user.

The joint was designed to be secured to the lateral side of the prosthetic socket and positioned at the anatomical hip joint axis of rotation. Since the available space on the lateral side of the socket can be limited for some amputees, the joint maintains a low-profile connection to the socket, and electrical components are positioned in the usable space beneath the prosthetic socket. This mounting orientation could achieve more symmetrical loading since the ground reaction force vector would not be in front of the socket.

The tensioning solution (Figure 3) required few components and, collectively, these parts did not protrude laterally beyond the geometrical requirements, which was desirable. However, assembly was awkward and re-tensioning took more time than might be available in a patient fitting session. Initially, there was a moderate amount of constructional slack due to the spliced connections at the end of each rope, requiring re-tensioning during the pilot trial. In future iterations, rope pre-loading procedures, alternative tensioning methods, or different rope terminations designs could be explored.

### Discussion of results

After mechanical testing at a weight rating of 100.0 kg, the LMPHJ showed no signs of failure or permanent deformation. While the settling force duration was not recorded for the ML test (Figure 12), this was acceptable because static testing was evaluating the proof and failure level tests loads. Applied loads and displacements were highly correlated, with the largest displacements occurring during the ML test, likely due to the joint geometry being less resistant to bending in the corresponding test plane. The lower deflection values shown in Figure 12 between 5 s and 10 s of each test were likely a result of the programmed limits of the testing machine. The initial loads measured for each test were 70–85 N, which were greater than the 50 N stabilizing force that was expected. Therefore, to achieve the force specified in the program, the machine needed to unload the joint from its original position, changing displacement direction. Comparison of initial and final joint positions revealed that there was no permanent deformation.

Functional testing demonstrated that the LMPHJ enables walking over level ground. Three participants could walk using the hip prosthesis simulator for training and testing sessions. An average stride time of 2.35 +/− 0.06 s (Table 2), maximum flexion of 51.14 +/− 3.43°(Table 3), and a maximum extension of 24.53 +/− 4.33° were achieved (Table 3). Hip extension is blocked by a mechanical stop for passive hip joints, to enable safe stance phase, so the powered hip enables people to use their preferred hip extension and thereby stride length when walking.

Since the gait profile (Figure 11) implemented by the simple control system was consistent for each test session, a similar stride time and cadence was measured across all participants (i.e., pilot test participants learned to walk at the pace set by the control pattern). One participant demonstrated equal step times between the prosthetic and intact limbs, indicating that the LMPHJ could provide good gait symmetry. Based on subjective feedback, the participants perceived that the LMPHJ required less energy and pelvic tilt to walk when compared to their previous experience with the Helix^3D^ on the simulator. Additional testing will be required to quantify these claims.

## Conclusion

A novel laterally mounted powered hip joint was designed and validated to provide a functional solution for amputees who use a HKAF prosthesis. The new LMPHJ is designed to promote natural gait patterns and offer safe and controlled movement across various daily activities. A full-scale prototype was designed, fabricated, and assembled with machined and commercially available components. Mechanical tests, based on existing international standards, were conducted with success. LMPHJ functionality and usability were assessed analytically and subjectively using a simulator that enabled able-bodied participants to walk with the powered hip prosthesis. Validation was completed by evaluating the design against the original requirements, in which the joint fully met each one.

The LMPHJ meets the mechanical requirements for level ground walking, stairs, and inclines with the powered hip. Functional testing across these activities will require a complete control system that adapts to the user’s walking style, which is currently being implemented and assessed. Functional testing indicated that the LMPHJ enabled safe walking with a prosthesis simulator. While participants perceived that the LMPHJ required less energy and pelvic tilt to walk when compared to their previous experience with the Helix^3D^, additional testing with amputee participants will be required to quantify these observations.

Future research should include implementing an outer casing to enclose all moving components, optimizing system weight, improving rope, or tensioning design, and functional testing with hip-level amputees including biomechanical kinematic and kinetic motion analysis.
